# Ouabain Induces Transcript Changes and Activation of RhoA/ROCK Signaling in Cultured Epithelial Cells (MDCK)

**DOI:** 10.3390/cimb45090475

**Published:** 2023-09-14

**Authors:** Jacqueline Martínez-Rendón, Lorena Hinojosa, Beatriz Xoconostle-Cázares, José Abrahán Ramírez-Pool, Aída Castillo, Marcelino Cereijido, Arturo Ponce

**Affiliations:** 1 Department of Physiology, Biophysics and Neurosciences, Centro de Investigación y Estudios Avanzados del Instituto Politécnico Nacional, Ciudad de Mexico 07360, Mexico; jamare@fisio.cinvestav.mx (J.M.-R.); lorena.hinojosa@cinvestav.mx (L.H.); aida.castillo@cinvestav.mx (A.C.); cereijido@cinvestav.mx (M.C.); 2 Molecular Medicine Laboratory, Unidad Académica de Medicina Humana y C.S., Campus UAZ Siglo XXI-L1, Universidad Autónoma de Zacatecas, Zacatecas 98160, Mexico; 3 Department of Biotechnology and Bioengineering, CINVESTAV-IPN, Ciudad de Mexico 07360, Mexico; bxoconos@cinvestav.mx (B.X.-C.); jramirezp@cinvestav.mx (J.A.R.-P.)

**Keywords:** ouabain, gene expression, epithelia, cell–cell contacts, MDCK, MYO9A, Na^+^/K^+^-ATPase

## Abstract

Ouabain, an organic compound with the ability to strengthen the contraction of the heart muscle, was originally derived from plants. It has been observed that certain mammalian species, including humans, naturally produce ouabain, leading to its classification as a new type of hormone. When ouabain binds to Na^+^/K^+^-ATPase, it elicits various physiological effects, although these effects are not well characterized. Previous studies have demonstrated that ouabain, within the concentration range found naturally in the body (10 nmol/L), affects the polarity of epithelial cells and their intercellular contacts, such as tight junctions, adherens junctions, and gap junctional communication. This is achieved by activating signaling pathways involving cSrc and Erk1/2. To further investigate the effects of ouabain within the hormonally relevant concentration range (10 nmol/L), mRNA-seq, a high-throughput sequencing technique, was employed to identify differentially expressed transcripts. The discovery that the transcript encoding MYO9A was among the genes affected prompted an exploration of whether RhoA and its downstream effector ROCK were involved in the signaling pathways through which ouabain influences cell-to-cell contacts in epithelial cells. Supporting this hypothesis, this study reveals the following: (1) Ouabain increases the activation of RhoA. (2) Treatment with inhibitors of RhoA activation (Y27) and ROCK (C3) eliminates the enhancing effect of ouabain on the tight junction seal and intercellular communication via gap junctions. These findings further support the notion that ouabain acts as a hormone to emphasize the epithelial phenotype.

## 1. Introduction

Ouabain, a noteworthy organic chemical compound [1], has garnered significant attention due to its diverse range of toxic, therapeutic, and physiological effects on various animal species, including humans. It falls into a category of chemical compounds known as cardiac glycosides [2,3]. Originally obtained from plants and subsequently isolated [4], ouabain was once utilized as a medicinal treatment for improving heart conditions. Its ability to enhance the contraction of the cardiac muscle was particularly beneficial. However, due to its narrow therapeutic window, where the effective dose is very close to the toxic dose, the use of ouabain in congestive heart failure has nearly ceased [5].

At the molecular level, ouabain is known to interact with the sodium–potassium ATPase (Na^+^/K^+^-ATPase), resulting in the inhibition of its pumping capacity at concentrations in the micromolar range in most animal species. This inhibition accounts for its toxic and lethal effects, considering the widespread presence of this pump in all animal cells [6]. However, when administered at controlled doses, ouabain induces inhibition of Na^+^/K^+^-ATPase, leading to what is known as a positive inotropic effect, which explains its properties in cardiac tissue [7,8].

In addition to its inhibitory effect on Na^+^/K^+^-ATPase, it has been observed that at picomolar to nanomolar doses, ouabain (as well as other cardiac glycosides) activates Na^+^/K^+^-ATPase to function as a receptor and transducer. This activation initiates one or more signaling cascades, resulting in physiological effects in various tissues [9,10]. Interestingly, it has been shown that some animal species, including humans, produce, by themselves, a chemical compound undistinguishable from plant-derived ouabain, which is produced and secreted from the adrenal glands and released into the bloodstream in concentrations in the peak to the nanomolar range [11]. Hence, ouabain has been considered as a new hormone [12,13,14,15], of which, however, its physiological functions are mostly unknown.

We investigated the effects of ouabain at hormonally relevant concentrations (in the nanomolar range) on epithelial physiology. To study these effects, we employed MDCK (Madin–Darby canine kidney) cells as a biological model since they form monolayers in culture that exhibit typical epithelial properties [16,17]. Our research has demonstrated that ouabain influences various important biological aspects of epithelial cells related to cell-to-cell contact and apical/basolateral membrane polarity. Specifically, we found that ouabain: (1) enhances intercellular contact mediated by tight junctions [18]; (2) induces changes in adherens junctions by promoting increased expression of E-cadherin, β-catenin, and γ-catenin in the cell membrane [19]; (3) enhances gap junctional communication between cells arranged in mature epithelial monolayers [20] by modulating the membrane distribution of Cx32 and Cx43 [21]; and (4) accelerates the reestablishment of the epithelial phenotype in cells seeded at confluency after trypsinization, as evidenced by the expedited expression of an apical cilium [22]. Moreover, our investigations have revealed that ouabain affects various processes and that the Na^+^/K^+^-ATPase acts as the receptor responsible for initiating signaling cascades upon binding to ouabain. These cascades involve the participation of cSrc and Erk1/2 in their initial stages. However, the involvement of other downstream components and the potential inclusion of additional signaling pathways remain to be elucidated. Based on these findings, we formulated a hypothesis suggesting that one of the physiological roles of ouabain is to enhance the epithelial phenotype by modulating multiple processes associated with intercellular contact and epithelial polarity [23,24]. In addition to its direct modulation of diverse biological processes, ouabain has been reported to induce the nuclear relocation of signaling Nuclear Adhesion Complexes (NACos) [25], as well as β-catenin, a peripheral protein involved in adherens junctions [19]. These results suggest that ouabain, within the nanomolar range, may also induce changes in gene expression in epithelial cells.

With the aim of enhancing our understanding of the physiological and molecular shifts induced by ouabain in epithelial cells, particularly concerning intercellular connections, we employed mRNA-seq, a high-throughput sequencing technique often referred to as next-generation sequencing (NGS) [26,27]. This study aimed to ascertain whether ouabain, administered at a physiological concentration of 10 nmol/L, triggers any changes in the transcriptome of MDCK epithelial cells. If such changes occur, our goal is to pinpoint the specific genes associated with the modulation of intercellular communication.

As described below, the preliminary findings of our study led us to hypothesize and subsequently investigate the involvement of Rho-A and its downstream effector ROCK in the signaling pathways activated by ouabain to modulate key cell-to-cell contacts in epithelial cells, including tight junctions and gap junctions.

## 2. Materials and Methods

### 2.1. Cell Culture

A vial stored in liquid nitrogen of MDCK cells, purchased from the American Type Culture Collection (ATCC) (Manassas, VA, USA), was thawed and seeded in tissue culture flasks and grown in a 5% CO_2_ and humidified atmosphere at 36.5 °C, in DMEM (Dulbecco’s Modified Eagle Medium, Cat. 12100-061, GIBCO, Grand Island, NY, USA), supplemented with 10,000 U/μg/mL penicillin-streptomycin (In Vitro, Cat. A-01, Roma Sur, Mexico), and 10% FBS (fetal bovine serum, Cat. 160000-44, GIBCO, Grand Island, NY, USA), this medium is hereafter referred to as CDMEM. For all experimental assays, cells were harvested with trypsin (in vitro, Cat. EN-005, Roma Sur, Mexico), seeded at confluency (1–2 × 10^5^ cells/cm^2^), in a well of a 24-well plate (Costar, Cat. 3524, Corning, NY, USA), which may have contained a glass coverslip or transwell, as required by the experiments. To obtain mature monolayers, cells were seeded at confluency and incubated in CDMEM for 24 h, then washed with PBS (phosphate-buffered saline, Cat. 21300-058, GIBCO, Grand Island, NY, USA) and incubated in DMEM supplemented with 1% FBS for an additional 24 h, before treatments.

### 2.2. Massive RNA Sequencing (mRNA-Seq)

#### 2.2.1. Treatment of MDCK Cells in Mature Monolayers with or without Ouabain

Cells in mature monolayers were incubated with 10 nmol/L ouabain as follows: DMEM fresh medium supplemented with FBS was mixed with ouabain and used to replace the medium, and cells were incubated for 1 h at 37 °C. All samples were placed in 15 mL falcon tubes containing 10 mL of RNA stabilization solution ((RNAlaterTM; Cat AM7021, Thermofisher, Whaltham, MA, USA). Samples were stored at 4 °C for further analysis.

#### 2.2.2. RNA Extraction

The culture medium was removed, and 5 mL of RNA stabilization solution (RNAlaterTM; Cat AM7021, Thermofisher, Whaltham, MA, USA) was added to the flask. Monolayered cells were scraped using a plastic rod and then deposited into a sterile tube. RNA extraction was performed using the total RNA from the blood and tissues kit (Biopure, Mexico) and resuspended in 30 µL RNase-free water. RNA concentration and 260/280 nm ratio were determined with a Nanodrop One spectrophotometer (Thermo Scientific; Waltham, MA, USA). The RNA integrity was verified using denaturing agarose electrophoresis, and visualization was carried out with a BioDocAnalyze gel documentation system (Biometra Gmbh, Jena, Germany).

#### 2.2.3. Library Preparation and Sequencing for Massive RNA Sequencing (RNA-Seq)

RNA from three independent flasks treated with ouabain were pooled, as well as RNA of control, untreated cells. Therefore, each sample consisted of three biological replicates. RNA was purified as described and then treated with DNase I (Invitrogen, Carlsbad, CA, USA). Each sample was diluted to a final concentration of 0.13 µg/µL, and one volume (15 µL) of RNA stabilizing agent (RNAstat; Biopure, Mexico) was added. The samples were sent to Otogenetics^©^ Corporation (Atlanta, GA, USA) for sequencing. The RNA-seq method used was based on poly (A+) selection, which enriches eukaryotic mRNA and other polyadenylated RNAs. Each selected sample was processed with the TruSeq RNA Sample Preparation Kit (Illumina, San Diego, CA, USA), and index codes were assigned to identify each sample independently. RNA sequencing libraries were generated using Illumina sequencing instrumentation (Illumina HiSeq2500). Multiplexed libraries were sequenced on a single flow cell of the HiSeq2500 platform to generate 125 bp paired-end reads. Each RNA-seq set consisted of 40 million reads per sample.

#### 2.2.4. Bioinformatic Analysis

Data processing was carried out as follows: quality of sequence reads was assessed using the FASTQC program. Prior to assembly, sequences were processed with a quality control script (https://github.com/Czh3/NGSTools/blob/master/qualityControl.py, accessed on 1 July 2023) using the parameters −q 20, −p 90, and −a 30 to obtain high-quality paired sequences. SeqPrep v1.1 (https://github.com/jstjohn/SeqPrep, accessed on 1 July 2023) was used to merge overlapping paired reads with a minimum of 87% coincidence required in the overlapping region. High-quality merged and unmerged reads were de novo assembled with Trinity 2.4.0 [28] using default parameters. The resulting contigs (unigenes) were processed with Deconseq [29] to eliminate contaminating sequences. AlignWise was used to identify open reading frames (ORFs) and their translated amino acid sequences. Insertions and deletions within and outside of ORFs were corrected [30]. Redundant sequences with more than 90% of identity between them were removed. Non-redundant sequences were obtained with BlastClust [31].

The resulting non-redundant translated sequences of the transcriptome were functionally annotated by assigning them the function of the highest match resulting from the alignment with BLASTp (https://blast.ncbi.nlm.nih.gov/Blast.cgi?PAGE=Proteins, ccessed on 1 July 2023) using an E-value threshold of 10-5. The reference canine genome was used as a model for further analyses (Genome GCA_000002285.4 of Canis familiaris). Global expression profiles were compared for each transcriptome set to identify differentially expressed genes employing DESeq2, with a *p*-value of 0.05, selecting those with a log2-fold change ≥ 2. High-quality (HQ) sequences were mapped to the reference transcriptome (unigenes) with the RSEM package [32]. This program uses short sequence mapping tools such as Bowtie2 and yields the normalized expression profile as TPM (transcripts per million) and FPKM (fragments per kilobases of contigs/genes per million mapped reads). The resulting counts were processed with the R/Bioconductor DESeq package [33], which normalizes samples by pairwise comparison using an algorithmic expression based on the hypothesis that most genes are not differentially expressed. 

### 2.3. Quantitative Reverse Transcription-Polymerase Chain Reaction (RT-qPCR)

Total RNA was isolated (extracted) from MDCK cells using the TRI-reagent (Invitrogen, Cat. 15596018, Waltham, MA, USA). RT-qPCR was performed by a one-step method employing the Power SYBR^®^Green qPCR kit (Applied Biosystems, Cat. 4389986, Waltham, MA, USA) according to the manufacturer’s instructions. Triplicate samples were subjected to qPCR by using the StepOne Real-Time PCR System (Applied Biosystems, Cat. 4376357, Waltham, MA, USA). qPCR conditions were: (1) an initial cycle of 5 min at 42 °C; (2) one cycle of 5 min at 92 °C; (3) 40 cycles of amplification (30 s at 92 °C and 30 s at 62 °C); (4) a melt curve (15 s at 95 °C); (5) 1 min at 60 °C; (6) 15 s at 95 °C. Primers for each target are shown in Table 1. Subsequent sequencing confirmed the identity of each amplicon. The results were calculated using the 2^−ΔΔCT^ method (Livak and Schmittgen, 2001) with the expression level of the endogenous PRP0 as the reference housekeeping control gene.

### 2.4. RhoA Protein Activation Assay

Cells were seeded at confluency (2 × 10^5^ cells/cm^2^) in a cell culture plate of a 6-well (Costar, Cat. 3516, Corning, NY, USA) and cultured in CDMEM for 24 h to form mature monolayers. Then, they were starved by incubation in depleted media (DMEM supplemented with 1% FBS) for 24 h before ouabain treatments. The level of GTP-loaded RhoA was measured with G-Lisa Assay Biochem Kit (Cytoskeleton, Cat. BK124, Denver, CO, USA) in samples derived from control and ouabain-treated cells (ouabain 10 nmol/L, 1 h). A total of 50 µg of protein was subjected to the G-LISA™ assay. Absorbance was read at 490 nm.

### 2.5. Transepithelial Electrical Resistance (TER)

The degree of TJ permeability to ionic solutes was assessed by measuring the TER of the cells grown on trans-well permeable supports. Monolayers were grown on trans well^®^ culture inserts (Costar, Cat. 3415, Corning, NY, USA) by seeding MDCK cells at a density of 1 × 10^5^ cells/cm^2^ and left to mature for 24 h and incubated in DMEM supplemented with 1% FBS for an additional 24 h before addition of treatments to media. TER was measured before and after 1, 2, and 3 days of ouabain treatment using an EVOM (Epithelial Voltameter; World Precision Instruments). Measurements are expressed as ohms per square centimeter (Ω·cm^2^).

### 2.6. Measurement of Gap Junctional Intercellular Communication by Dye Transfer Assays

Dye transfer assays consisted of impalement and injection, with glass micropipettes, of individual cells from mature monolayers grown on glass coverslips. Coverslips on which cell monolayers had been grown were placed in a translucent chamber filled with PBS plus Ca^2+^ (1.8 mM) solution at room temperature. Micropipettes were elaborated from borosilicate glass capillaries tubes (Kimax, cat 34500-99, Rockwood, TN, USA) on a vertical David-Kopf puller (DKI-700c, Tujunga, CA, USA). Those with a tip electrical resistance of 5–10 MOhms were backfilled with a saline solution containing 120 mM KCl, 5 mM NaCl, 1 mM MgCl_2_, 5 mM HEPES (pH 7.4), and Lucifer Yellow (Cat 67764-47-5, Sigma-Aldrich, St. Louis, MO, USA) (1%). After filling up, pipettes were attached to the holder device, which was mounted to a micromanipulator (PCS-750; Burleigh Instruments, NY, USA). For the impalement of cells, the chamber was mounted on the stage of an inverted microscope (Diaphot 300; Nikon, Tokyo, Japan) equipped with epifluorescence. Three independent trials were made. On each trial, a given number of repeats were made per coverslip. In each repeat, cells were randomly chosen from among those constituting the monolayer, then impaled and injected, one at a time, using a pneumatically driven microinjecting device (IM300; Narishige, NY, USA). After injections, the coverslips were rinsed with PBS and fixed by dipping into 4% paraformaldehyde, then rinsed (3×) with PBS and mounted using VECTASHIELD^®^ (H-1000; Vector Laboratories, Burlingame, CA, USA). Eight-bit images of the fluorescent cells were acquired at room temperature using a Zeiss M200 inverted microscope equipped with a Plan-NeoFluar 63× N.A. 1.25 objective lens, an AxioCam MRm camera, and software Axovision 4.8 (AXOVISION GmbH, Hanover, Germany). The captured images were imported into FIJI Is Just ImageJ software (release 2.8, NIH, Bethesda, MD, USA) to adjust the brightness and the contrast and GIMP (release 2.8.10, NIH) to compose the figures.

### 2.7. Materials and Chemicals

Ouabain (Sigma-Aldrich 11018-89-6) 1 mM stock was prepared in DMSO. Subsequent dilutions were made with PBS without Ca^2+^. Lucifer Yellow was obtained from Sigma-Aldrich (67764-47-5) and dissolved in a solution containing (100 mM KCl, 5 mM NaCl, 10 mM HEPES, 1 mM CaCl_2_, pH 7.4). C3 transferase from Clostridium botulinum (Sigma-Aldrich, Cat. CT3-A, St. Louis, MO, USA) was prepared as a 10 mM stock in water and used at a concentration of 1 μM. Y-27632 (Merck KGaA, Cat 688000, Darmstadt, Germany) was prepared as a 10 mM stock in water and used at a concentration of 1 μM.

### 2.8. Statistical Analysis

Statistical tests were performed with Prism, version 6 (GraphPad Software, San Diego, CA, USA). The results are expressed as the mean ± S.E. Statistical significance was estimated with a two-tailed Student’s *t*-test or one-way analysis of variance (ANOVA) followed by Bonferroni’s multiple comparison test (*: *p* < 0.05, **: *p* < 0.01, and ***: *p* < 0.001). All experiments were repeated at least three times. The number of independent experiments is represented by n.

## 3. Results

### 3.1. Ouabain Modifies Gene Expression in Epithelial Cells

**Figure 1:**
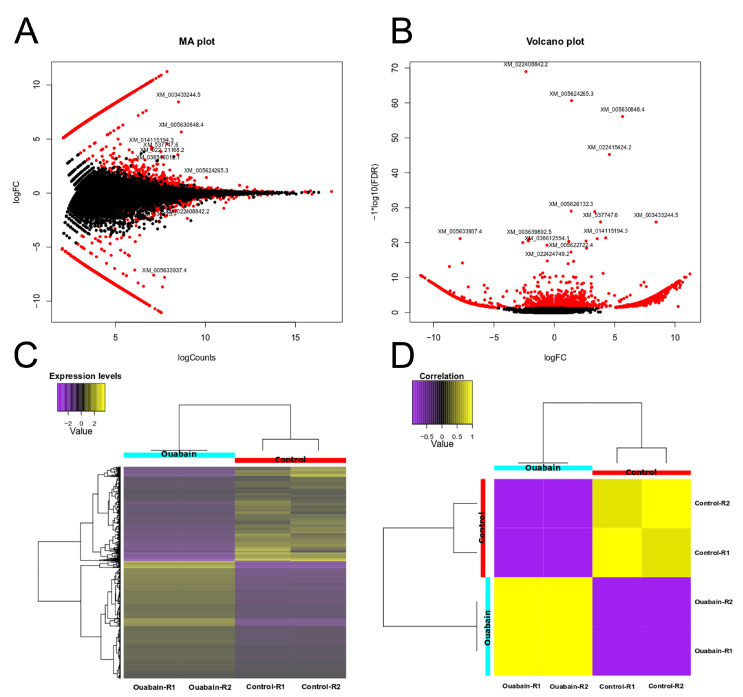
Differentially regulated transcripts by the effect of ouabain: (**A**) MA plot; (**B**) volcano plot; (**C**,**D**) heatmap indicating up and downregulated genes grouped according to their expression levels. Duplicate samples are shown. Transcripts in ouabain-treated MDCK cells are shown in left blue; control, untreated cells are indicated with red. Down-regulation is indicated with violet color, while up-regulation is shown in yellow according to color key present in (**D**).

Our initial aim was to investigate whether ouabain, at a concentration in the nanomolar range, causes transcriptional changes and, if so, identify those undergoing differential expression. To accomplish this objective, as outlined in Section 2 we employed the mRNA-seq technique to analyze and compare the transcriptomes of mature MDCK cell monolayers that were either treated with ouabain at a concentration of 10 nmol/L for 30 min, employing as control untreated MDCK cells. Figure 1A,B show the results in the form of an MA plot and a volcano plot. Figure 1C,D also show, in the form of a heatmap, the genes up- and down-regulated by treatment with ouabain. A more detailed version of Figure 1C, which show the list of genes included, is available as a supplement (Figure S1). Table 2 presents a compilation of ten transcripts that exhibited the most significant fold changes when subjected to ouabain treatment, encompassing both upregulated and downregulated genes.

MYDGF oversees the synthesis of myeloid-derived growth factor, a protein manufactured by monocytes and bone marrow-derived macrophages. This protein has exhibited cardioprotective and regenerative attributes after instances of myocardial infarction (MI) [34]. Its functionality revolves around stimulating the proliferation of cardiomyocytes through the activation of the c-Myc/FoxM1 pathway [35].

TM2D1 encodes for BBB, a β-amyloid binding protein that functions as a receptor for β-amyloid. This protein contains a G protein-coupling module and is associated with neuronal apoptosis, a well-known factor in the development of Alzheimer’s disease. There is evidence suggesting that the binding of β-amyloid to BBB triggers a G protein-regulated cell death program [36].

DCDX codes for a protein known as D-dopachrome decarboxylase-like or D-Dopachrome tautomerase, which primarily acts as an enzyme involved in melanogenesis, converting 2-carboxy-2,3-dihydroindole-5,6-quinone (D-dopachrome) into 5,6-dihydroxyindole [37,38]. Interestingly, DCDX shares similarities with macrophage migration inhibitory factor (MIF), a cytokine associated with inflammatory reactions and immune responses [39,40,41].

DCXR encodes for Dicarbonyl/L-xylulose reductase, an enzyme primarily responsible for converting L-xylulose into xylitol [42]. Recent observations suggest that DCXR may have a role in cell adhesion, potentially contributing to tumor progression and metastasis [43]. 

FH is the gene that codes for fumarate hydratase, an enzyme involved in the Krebs cycle, catalyzing the conversion of fumarate to L-malate [44]. In addition to its role in energy metabolism, FH has been implicated in the cellular response to DNA double-strand breaks and acts as a tumor suppressor [45]. Mutations in the FH gene are associated with hereditary renal cancer [46].

MRPL39 is a nuclear gene that encodes mitochondrial ribosomal protein L39, a component of the large subunit of the mitochondrial ribosome, which is essential for protein synthesis within mitochondria [47]. Dysregulation or mutations in MRPL39 can lead to mitochondrial dysfunction, associated with various human diseases such as metabolic disorders, neurodegenerative diseases, and cancer [48]. 

UBE4B encodes for Ubiquitin Conjugating factor E4B, a cytosolic E3/E4 ubiquitin ligase [49], which plays a role in linking ubiquitination and sorting mechanisms for the degradation of epidermal growth factor receptor (EGFR) [50]. Decreased expression of UBE4B is correlated with low cellular differentiation in neuroblastoma tumors [51]. In tumor cell lines, transient depletion of UBE4B results in increased levels of EGFR and activation of the downstream MAPK/ERK signaling pathway [52]. MTMR4 is the gene responsible for producing myotubularin-related protein 4 (also known as FYVE-DSP2), a dual specificity protein phosphatase containing the FYVE domain. MTMR4 dephosphorylates phosphatidylinositol 3-phosphate [53,54] and plays a role in regulating sorting from early endosomes [55]. 

UBTF codes for upstream binding transcription factor, RNA polymerase I (also nown as nucleolar transcription factor 1). It is involved in recognizing the ribosomal RNA gene promoter (TATA box) to activate RNA polymerase I transcription, working in cooperation with the transcription factor SL1/TIF-IB complex [56,57].

MYO9A is the gene responsible for encoding myosin-IXA, an unconventional type of myosin that possesses both motor and signaling functions, distinguishing it from conventional myosins. This distinction arises from a domain present in its tail region, which inactivates Rho (specifically isoforms A, B, and C) by converting its active, GTP-bound state to an inactive, GDP-bound state [58,59,60,61]. Due to its role in inhibiting Rho, myosin-IXA is considered a negative regulator of Rho [62].

**Figure 2:**
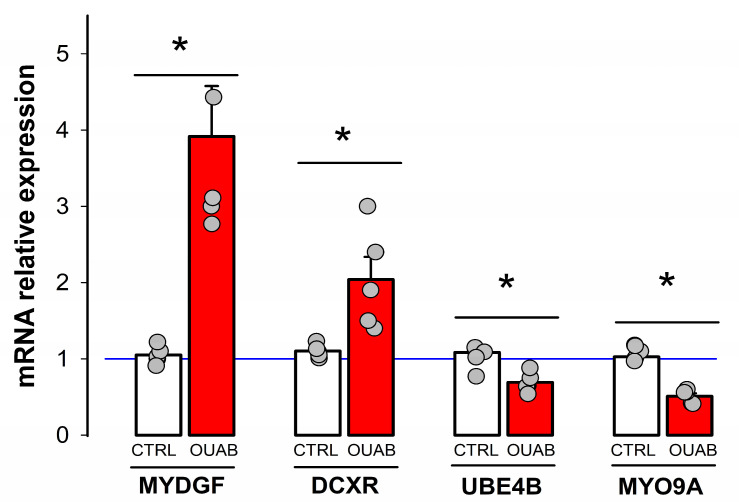
Validation of ouabain-induced differential expression of mRNA by RT-qPCR. The four pairs of bars show the average and standard error of the relative expression of mRNA in RT-qPCR assays of four different genes, which, according to the seq mRNA assay, had shown more variation. The grey circles denote the individual values obtained from 5 independent trials. The (*) symbol above the slash denotes a statistically significant difference (*p* < 0.05). Welch’s *t*-test, equal variances not assumed.

To validate these results, we assessed by quantitative RT-PCR (RT-qPCR) the expression of a set of selected transcripts. These included a pair of genes (MYDGF and DCXR), which increased its level of expression, and another (MYO9A and UBE4B), which decreased it. As shown in Figure 2, in all these cases, the results obtained with the RT-qPCR technique were correlated with those obtained by mRNA-seq. From these results, we therefore conclude that ouabain, in a concentration within the physiological range (10 nmol/L), effectively induces changes in the transcriptome of epithelial cells.

### 3.2. Downregulation of MYO9A Suggests the Participation of Rho/ROCK Signaling in the Modulation of Epithelial Properties by Ouabain

**Figure 3:**
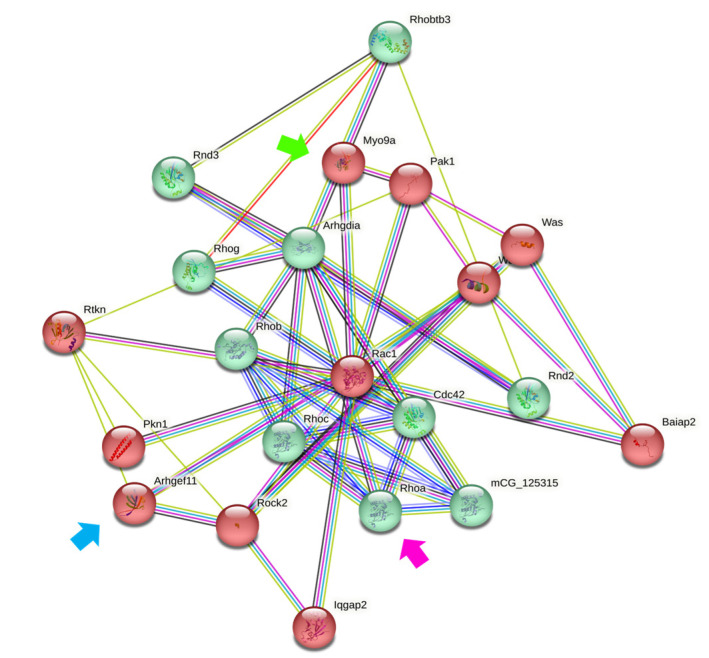
Functional protein association network (interactome) diagram of Myo9a. The interaction analysis of myo9a (*green arrow*) shows that there is evidence of interaction with recognized members of the Rho/ROCK signaling pathway, among which RhoA *(pink arrow)* stands out, and that it also includes upstream components such as Arhgef11 (*blue arrow*), a guanosine exchange factor. Lines of different colors indicate the type of evidence of the interaction. The green and red colors of the components indicate two levels of clustering. The permalink to access the diagram is: https://version-11-5.string-db.org/cgi/network?networkId=b8BzUXMHHAIQ, accessed on 11 May 2023.

Among the transcripts significantly altered by ouabain, MYO9A captured our interest due to its known involvement in biological processes related to intercellular connections and epithelial differentiation. In Table 3, an assembly of Gene Ontology (GO) terms [63,64] is presented, outlining MYO9A’s association with biological processes specific to epithelial structure, cell–cell interactions, and Rho signaling. Additionally, Figure 3 visually portrays the outcomes of computational interaction analysis carried out through the STRING Database (https://string-db.org/, accessed on 11 May 2023) [65]. This figure illustrates the connection of MYO9A (indicated by the green arrow) with various members of the Rho family, such as RhoA (depicted by the pink arrow), along with several Guanosine Exchange Factors (GEFs) (denoted by the blue arrow), pivotal components of the Rho/ROCK signaling pathway. These findings prompted us to contemplate the potential involvement of the Rho/ROCK signaling pathway in mediating ouabain’s effects on intercellular connections. 

### 3.3. Ouabain Induces a Temporary Reduction in the Amount of mRNA of MYO9A

**Figure 4:**
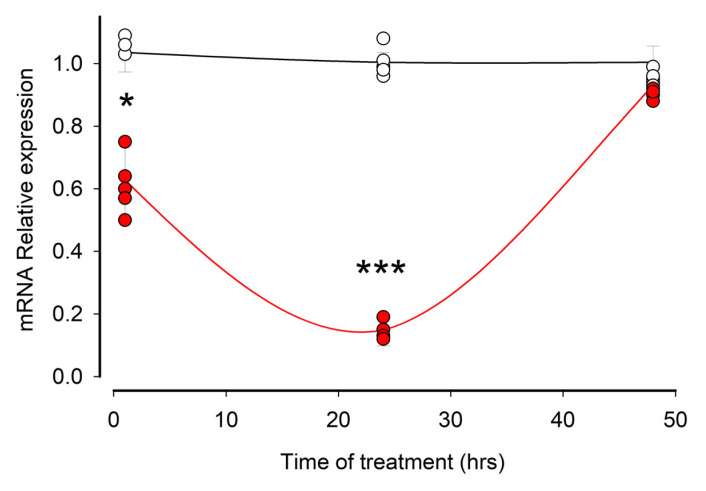
Ouabain induces a transient reduction in the amount of MYOA’s mRNA. Circles represent the averages (±S.E.) of the values estimated by RT-qPCR assays of the (standardized) amount of mRNA of MYO9A in the different times tested and are the result of 5 independent trials. The red circles correspond to the values obtained from ouabain treated cells, and the white circles are from untreated cells (control). The asterisks denote a statistically significant difference (*: *p* < 0.05; ***: *p* < 0.001). Student, *t*-test, equal variances assumed.

We first sought to determine how the amount of MYO9A’s mRNA changes over time of treatment with ouabain. Therefore, we employed RT-qPCR to measure the mRNA levels of MYO9a at longer time intervals (24 and 48 h) compared to the previously established validation period of 30 min for the mRNA-seq assay. Figure 4 illustrates the average relative expression values of MYO9A’s mRNA, obtained from cell samples subjected to ouabain treatment (represented by red circles) and untreated cells (represented by white circles) at various treatment durations, including the 30 min time point. As mentioned earlier, ouabain treatment initiates a decrease in the mRNA levels of MYO9A as early as 30 min. Following 24 h of treatment, ouabain further reduces the mRNA levels of MYO9A. However, after 48 h of ouabain treatment, this effect diminishes as the mRNA levels of MYO9A become indistinguishable from those observed in untreated cells.

### 3.4. Ouabain Enhances the Amount of GTP-Loaded RhoA

**Figure 5:**
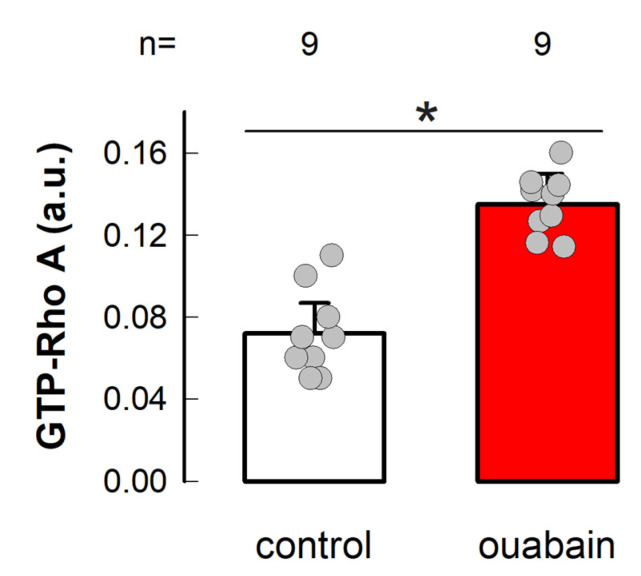
Ouabain promotes activation of RhoA. Bars are proportional to the average value (±S.E.) of the amount of GTP-loaded RhoA obtained by luminometry of 9 independent tests in which cells in mature monolayers were either treated with ouabain 10 nmol/L for 1 h (*red bar*) or not (*white bar*). (*) denotes a statistically significant difference (*p* < 0.05). The grey circles denote the individual values obtained in each case. Student, *t*-test, equal variances assumed.

Next, we assessed whether ouabain promotes the activation of RhoA. For this purpose, we used the reagents and procedures of a kit (G-LISA RhoA Activation Assay Biochem Kit, Cytoskeleton Inc., Denver, CO, USA), as detailed in Section 2, and estimated the values of GTP-loaded RhoA by luminometry measurements from samples obtained from cells in mature monolayers that were either untreated or treated with ouabain 10 nmol/L for 1 h. A treatment time that in previous studies has rendered a maximum effect [20]. As shown in Figure 5, treatment with ouabain effectively induced a statistically significant increase in the level of GTP-loaded RhoA.

The results mentioned earlier, which encompass the temporary decrease in MYO9A mRNA and the upsurge in RhoA-GTP levels, support the notion that Rho/ROCK are included in the signaling pathways by which ouabain modulates intercellular contacts in epithelial cells. These observations spurred us to delve into whether Rho/ROCK signaling is engaged in the two distinct biological processes previously recognized as being impacted by ouabain: the integrity of tight junctions and the communication through gap junctions.

### 3.5. Involvement of RhoA-ROCK on Ouabain-Promoted Enhancement of Tight Junction Sealness

In previous research reports, we demonstrated that ouabain leads to an increase in tight junction sealness (TJS) [18]. To investigate whether the Rho/ROCK pathway is involved in the signaling process through which ouabain affects TJS, we conducted experiments using two inhibitors: exoenzyme C3 transferase (C3), which selectively deactivates RhoA/B/C proteins through ribosylation [66,67], and Y-27632 (Y27), a potent inhibitor of ROCK1 (Ki = 140 nmol/L) and ROCK2 (IC50 = 800 nmol/L) [68,69].

To assess TJS, we measured the transepithelial electrical resistance (TER) of mature monolayers of MDCK cells. These cells were either treated with ouabain alone (10 nmol/L) or in combination with a 1 h pretreatment of either Y27 (1 µM) or C3 (1 µM). We obtained the average values from three independent trials, each consisting of five repetitions for each combination and treatment time point (0, 24, 48, and 72 h).

**Figure 6:**
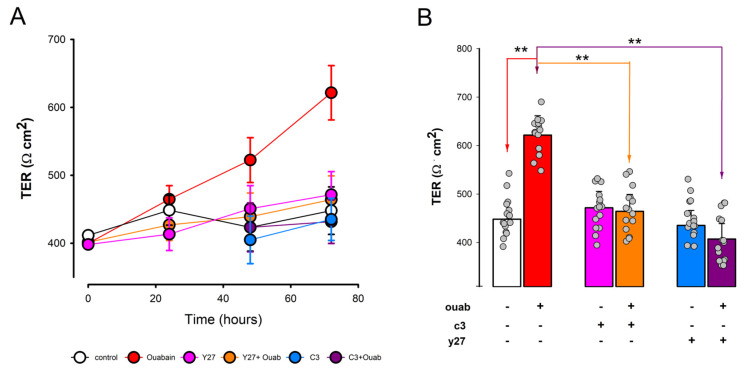
Inhibition of RhoA and ROCK activation abolishes the enhancement of TJS induced by ouabain. (**A**) Comparison of the time course of TER as a function of treatment time with none (white circles) ouabain (red circles), Y27 (purple circles), C3 (blue circles), Y27 + ouabain (orange circles), or C3 + ouabain (purple circles). (**B**) Comparison and statistical significance of abolishment of the enhancing effect on TJS induced by ouabain caused by C3 and Y27 after 72 h of treatment. TER values are the average (±S.E.) of 15 replications, 5 from each of three independent trials. The grey circles denote the individual values obtained on each trial. (**) denotes a statistically significant difference with *p* < 0.005. Welch’s *t*-test, equal variances not assumed.

Figure 6A shows the mean values (±S.E.) or TER for each experimental combination at each treatment time point. It is evident that ouabain induces a significant increase in TER, which persists for up to 72 h of treatment. In contrast, the control (no treatment) and the treatments with either Y27 or C3 show no significant changes over time. Figure 6B compares the average TER values of each combination after 72 h of ouabain treatment. The average TER value for monolayers treated with ouabain at 10 nmol/L for 72 h (621.6 ± 40.0 Ω∙cm^2^, n = 12) is significantly higher (*p* > 0.005) than that of untreated cells (448.0 ± 35.0 Ω∙cm^2^, n = 12). Treatment with either C3 or Y27 alone does not result in significant changes compared to the untreated cells. However, the value of TER in monolayers treated with ouabain + C3 (431.7 ± 32.0 Ω∙cm^2^, n = 12) was significantly lower (*p* < 0.005) than that obtained from monolayers treated with ouabain only. Likewise, the value obtained from monolayers treated with ouabain + Y27 (464.2 ± 35.0 Ω∙cm^2^, n = 12) was significantly lower (*p* < 0.005) than that obtained from monolayers treated with ouabain. From these results, it follows that both C3 and Y27 effectively abolished the enhancement of TJS induced by ouabain, which in turn supports the hypothesis that RhoA/ROCK are included in the signaling cascade by which ouabain acts on TJS.

### 3.6. Involvement of RhoA-ROCK on Ouabain-Promoted Enhancement of Gap Junctional Intercellular Communication

To assess the extent of gap junctional intercellular communication (GJIC), we conducted GJIC dye transfer assays following the methodology outlined elsewhere [20] and described in detail in Section 2. This procedure involves randomly selecting a cell from a mature monolayer and injecting it with lucifer yellow, which, if there is a gap junctional communication, will eventually diffuse to neighboring cells. The average number of stained cells per injection serves as an estimation of GJIC intensity. To obtain reliable results, we performed multiple repetitions for each experimental combination, both with and without treatment involving ouabain, as well as pretreatment with C3 or Y7. Like TJS analysis, we conducted statistical comparisons of the values obtained for each experimental combination. In this case, the treatment with ouabain lasted only one hour, as previous research demonstrated that this duration yields the maximum intensity of GJIC induced by ouabain [20].

To investigate the involvement of RhoA and/or ROCK in the signaling pathway through which ouabain affects intercellular junctional gap communication (GJIC), we conducted experiments to determine if the inhibitors C3 and Y27 would eliminate the enhancement of GJIC induced by ouabain, as previously reported [20].

**Figure 7:**
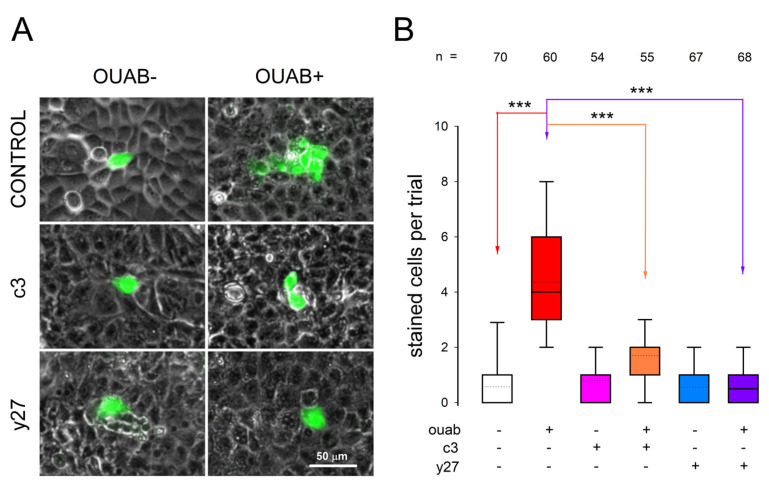
Inhibition of RhoA and ROCK activation abolishes the enhancement of GJIC induced by ouabain. (**A**) Phase contrast images of representative dye transfer trials of each of the different treatments to probe the effect of C3 and Y27 with or without ouabain. The green color is from staining with lucifer yellow. (**B**) Box plot shows the abolishment of the enhancing effect of GJIC induced by ouabain, caused by Rho and ROCK inhibitors C3 and Y27. Continuous and dotted lines inside boxes denote mean and median of number of cells stained. The number above each bar indicates the number of trials. The combination treatments are indicated in the below boxes. (***) denotes a statistically significant difference (*p* < 0.0001). Multiple comparisons versus control group (Holm–Sidak method).

Figure 7A displays representative images of the dye transfer trials under each experimental condition, while Figure 7B presents a comparison of the average number (±S.E.) of stained cells per trial for each experimental combination.

As observed in Figure 7A, the addition of ouabain at a concentration of 10 nmol/L to the extracellular medium for a duration of 1 h leads to a significant increase in the average number of stained cells compared to the corresponding value obtained from untreated control cells. The addition of only C3 or Y27 does not result in a statistically significant change compared to the control cells. However, when combined with ouabain, the addition of C3 significantly reduces the response observed with ouabain alone. Similarly, treatment with Y27 causes a significant reduction in the enhancement produced by ouabain alone. 

These findings indicate that, like the modulation of TJS, the signaling cascade activated by ouabain to regulate GJIC involves the participation of RhoA/ROCK.

## 4. Discussion

Although ouabain has been utilized by humans for centuries, our understanding of its properties has significantly evolved in recent decades. Originally considered to be a poison, it is now regarded as a hormone with relatively limited knowledge about its various roles. In addition to its well-documented cardiotonic effects, ouabain has been associated with numerous physiological processes of significant importance. Notably, its influence on fundamental biological processes such as proliferation, viability, and cell differentiation has sparked interest in further exploring the molecular components and mechanisms it activates, both in normal physiological states and pathological conditions like cancer. As previously described, ouabain exerts its diverse effects by interacting with Na^+^/K^+^-ATPase, which serves a dual function as both a pump and a receptor. Depending on the concentration, ouabain can inhibit the pumping activity of Na^+^/K^+^-ATPase or act as a receptor, leading to the activation of various signaling pathways [70,71].

In this study, our first purpose was to investigate whether ouabain in a concentration that does not inhibit the pumping capacity of Na^+^/K^+^-ATPase can induce changes to the transcriptome of epithelial cells (MDCK) organized as mature epithelial monolayers, and if so, to be able to identify novel signaling pathway components, besides those already described (cSrc and Erk 1/2), which modulates intercellular contacts in epithelial cells [18,19,20,21,22,23,24]. 

As described above, we found that treatment with ouabain 10 nM for 30 min caused a relatively moderate effect on the transcriptome, both in terms of the number of transcripts affected and the magnitude of their fold change. Among the transcripts that underwent noteworthy alterations, there were no initial indications of any candidates involved in the regulation of intercellular connections or signaling. Nevertheless, the inclusion of MYO9A in these modified transcripts prompted us to formulate and subsequently investigate the notion that Rho and/or ROCK might be implicated in the signaling cascade through which ouabain brings about changes in tight junction or gap junction connections. The subsequent confirmation that the application of Rho inhibitors and ROCK abolishes the modulating effect of ouabain on both TER and GJIC lends support to this hypothesis. These findings hold significance as they broaden our understanding of the constituents that form the signaling pathways prompted by ouabain. Furthermore, they corroborate our overarching theory that one of ouabain’s physiological roles is to accentuate the epithelial phenotype.

Nevertheless, it is important to note that these deductions are presently restricted to ouabain-dependent modulation of tight junctions and gap junctions. This is because, up to this point, we have not assessed whether Rho/ROCK also partake in the modification of other attributes and processes influenced by ouabain, such as the adjustment of apical-basolateral polarity, cilliogenesis, or the regulation of ion channel expression.

Besides our findings, there are additional accounts detailing ouabain’s capacity to induce alterations in transcript expression. However, these instances pertain to diverse cell types and alternate experimental conditions. For instance, it has been observed that in quiescent vascular smooth muscle cells, exposure to 1 mM ouabain prompts the activation of early response genes like c-Fos and c-Jun [72]. In normal rat kidney epithelial cells (NRK), a 3-h treatment with 1 mM ouabain heightened mRNA levels of Na^+^/K^+^-ATPase α1 and β1 subunits [73]. Similarly, in cerebellum granule cells, a 3 h exposure to 1 mM ouabain triggered increased mRNA levels of genes involved in intracellular signaling and transcription regulation, including Npas4, Fos, Junb, Atf3, and Klf4 [74]. Likewise, in neuron-like cells (PC12), stimulation with 100 nM ouabain hampers cell growth while elevating the expression of proto-oncogenes c-myc and c-fos [75]. Furthermore, treatment of neonatal rat cardiac myocytes with 100 µM ouabain results in heightened mRNA levels of c-fos, c-jun, and the transcription factor AP-1 [76]. It is notable that the concentrations employed to investigate ouabain’s effects in these references vastly exceed the concentration utilized in our study. Furthermore, several of these reports attribute the capacity to induce shifts in gene expression to an imbalance in intracellular Na^+^ and K^+^ concentrations [72,77]. Therefore, the role of ouabain in these cases is attributed to the inhibition of Na^+^/K^+^ ATPase’s pumping function rather than its activation as a receptor.

Our results lend more support to the notion that ouabain activates the receptor function of Na^+^/K^+^ ATPase because the concentration of 10 nM does not cause inhibition of the Na^+^/K^+^ ATPase [78]. Additional reports have also documented that ouabain, when present in nanomolar concentrations, induces modifications within the transcriptome. A recent investigation, for instance, uncovered that subjecting human pluripotent stem cell-derived neurons to 30 nM ouabain led to changes in gene expression, involving the activation of MAP-kinase ERK1/2 as well as the transcription factor cAMP response element-binding protein (CREB) [79]. While the ouabain concentration employed in this study (30 nmol/L) resembled that of our own (10 nmol/L), notable disparities in outcomes emerged, particularly regarding the quantity and diversity of affected genes. It is important to acknowledge that a direct comparison is inherently limited due to variations in cell types (neurons versus epithelial cells) and treatment durations (16 h versus 1 h).

On the other hand, in relation to the findings of the mRNA-seq assay, a more comprehensive examination of the significance of the modified transcripts could potentially provide deeper insights into additional biological processes influenced by ouabain. Some of these processes, such as cell viability, apoptosis, and migration, have thus far remained beyond the focus of our investigation. Consequently, detailing the transcripts subject to alteration by ouabain serves as a valuable point of reference for enriching our comprehension of the diverse physiological mechanisms that ouabain elicits within epithelial cells.

**Figure 8:**
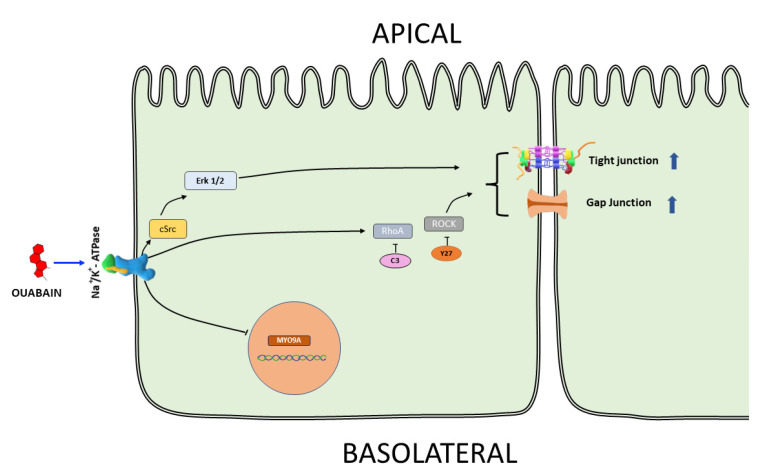
Schematic representation of the main findings of this work. Short-term treatment with ouabain 10 nmol/L causes a decreased content of MYO9A mRNA. The fact that Y27 and C3 inhibited ouabain-induced enhancement of GJIC and TJS indicates participation of Rho-A and ROCK in the signaling cascade activated.

In summary, as depicted in Figure 8, this study has provided evidence that ouabain, administered at a dose consistent with its hormonal and physiological range, triggers alterations in the transcriptome of MDCK cells organized as a confluent monolayer. These alterations include a reduction in the mRNA levels of MYO9A. Concurrently, ouabain incites modifications in intercellular connections, leading to heightened communication through gap junctions and enhanced integrity of tight junctions. The sequence of activation events responsible for these changes involves the participation of RhoA/ROCK signaling.

## 5. Conclusions

Exposing mature monolayers of MDCK epithelial cells to a 10 nM ouabain treatment for 30 min. results in alterations to the transcriptome. 

The signaling cascade triggered by ouabain to regulate cell-cell interactions, specifically tight junctions and gap junctions, involves the involvement of Rho and ROCK.

## Figures and Tables

**Table 1:** Primers for RT-qPCR assays.

MYDGF	forward	5′-CTTGTGGGCTGCGTTGCTGCTG-3′
	reverse	5′-GACTCATCTGCCACTTCTCGTTGG-3′
MYO9A	forward	5′-CTTCGAGCTATGGGCCTTCAGGAG-3′
	reverse	5′-GTAGTGGGTCAATGGTGTCAGGGC-3′
DCXR	forward	5′-GCGTCTACTGCTCCACCAAGGGTG-3′
	reverse	5′-GGTCACTCAGCAGGAAGAGGATGG-3′
UBE4B	forward	5′-CATGTCCCAGGTGGATGTGGATTC-3′
	reverse	5′-CTGTCTGGTGACCGAGCTGCTGC-3′
PRP0	forward	5′-GCAGGTGTTTGACAATGGCAGC-3′
	reverse	5′-GCCTTGACCTTTTCAGCAAGTGG-3′

**Table 2:** List of genes whose expression is modified by treatment with ouabain 10 nmol/L, as revealed by mRNA-seq.

Number	Gene	Description	Acc. No.	Fold Change
1	MYDGF	Myeloid derived growth factor	XM_849589	4.53
2	TM2D1	TM2 domain-containing protein 1-like, transcript variant X2	XM_003639021	3.61
3	DCDX	D-dopachrome decarboxylase-like	XM_003639927.2	2.99
4	DCXR	Dicarbonyl/L-xylulose reductase	XM_038675393.1	2.62
5	FH	Fumarate hydratase transcript variant 1	XM_537215.4	2.60
6	MRPL39	Mitochondrial ribosomal protein L39 transcript variant X2	XM_005638779.1	2.07
7	UBE4B	Ubiquitination factor E4B transcript variant X1	XM_844731.3	0.43
8	MTMR4	Myotubularin related protein 4 transcript variant X5	XM_005624734.1	0.32
9	UBTF	Upstream binding transcription factor, RNA polymerase I	XM_537622.5	0.19
10	MYO9A	Myosin IXA	XM_544755.4	0.16

**Table 3:** List GO terms describing biological processes for MYO9A related to epithelial morphology cell–cell contacts and Rho signaling (accessible through http://geneontology.org/, accessed on 11 May 2023).

Biological Process	GO-Term
Apical junction assembly	GO:0043297
Cell adhesion	GO:0007155
Cell junction assembly	GO:0034329
Cell–cell junction organization	GO:0045216
Epithelial cell differentiation	GO:0030855
Epithelium development	GO:0060429
Establishment of epithelial cell apical/basal polarity	GO:0045198
Establishment or maintenance of cell polarity	GO:0007163
Morphogenesis of an epithelium	GO:0002009
Rac protein signal transduction	GO:0016601
Regulation of cell-substrate adhesion	GO:0010810
Rho protein signal transduction	GO:0007266

## Data Availability

Not applicable.

## Supplementary Materials

The following supporting information can be downloaded at: https://www.mdpi.com/article/10.3390/cimb45090475/s1. Figure S1. A more detailed version of the Heatmap figure shown in the manuscript (Figure 1D), which depicts the acccesion name of all genes whose expression level was modified by ouabain. Including upregulated (gold) and downregulated (purple).
