# Supplementary material for: Ouabain Induces Transcript Changes and Activation of RhoA/ROCK Signaling in Cultured Epithelial Cells (MDCK)
Source: Curr Issues Mol Biol. 2023 Sep 14;45(9):7538–56. doi: 10.3390/cimb45090475 (PMC10528288; doi:10.3390/cimb45090475)
Supplement: Supplementary file 1 [file cimb-45-00475-s001.zip › cimb-2555230-supplementary.pdf]

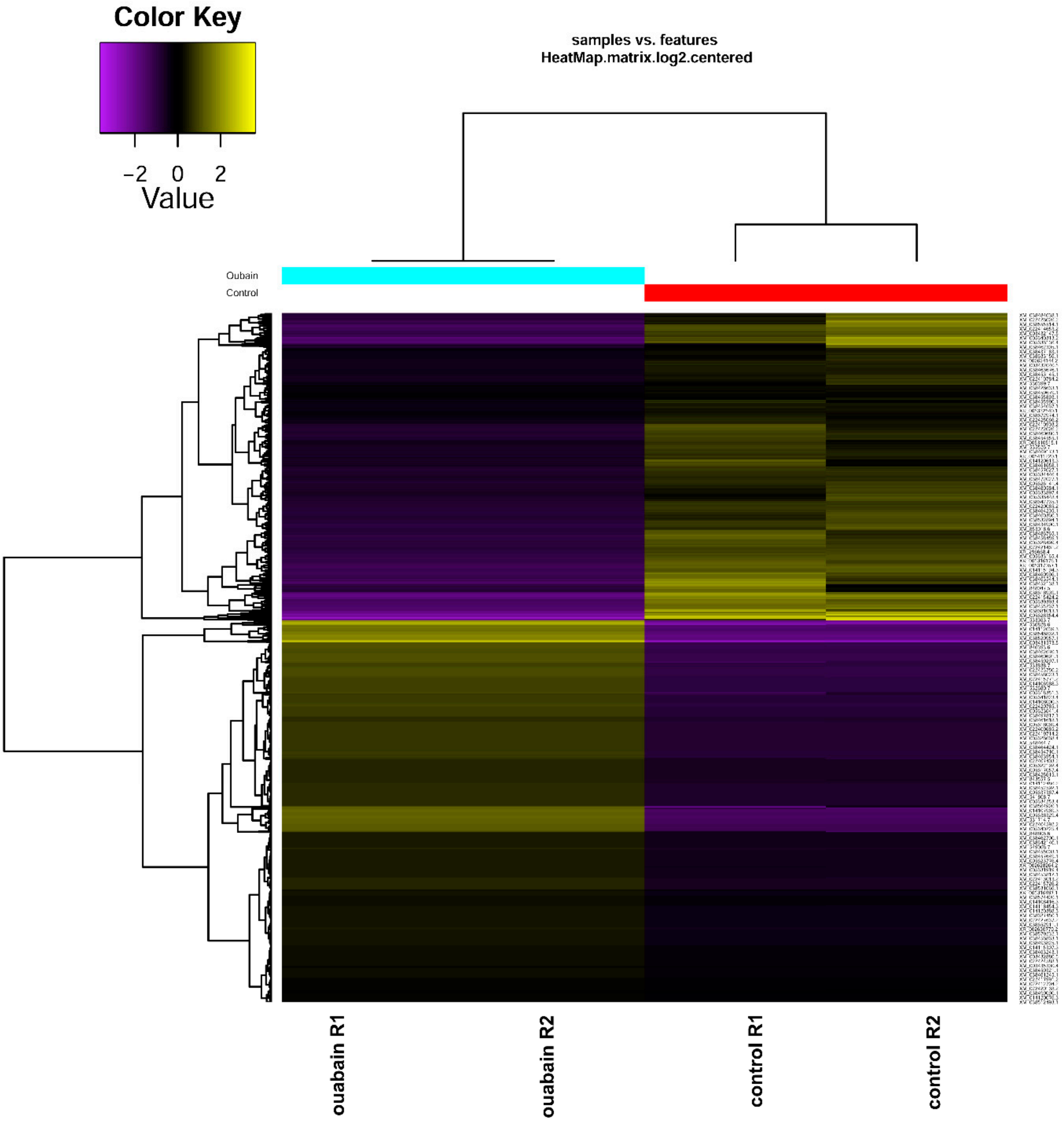
Color Key
−2 0 2
Value
samples vs. features
HeatMap.matrix.log2.centered
Oubain
Control
ouabain R1
ouabain R2
control R1
control R2

–

Figure S1. A more detailed version of the Heatmap figure shown in the manuscript (Figure 1D), which depicts the acccesion name of all genes whose expression level was modified by ouabain. Including upregulated (gold) and downregulated (purple).
